# Preexposure to Olive Oil Polyphenols Extract Increases Oxidative Load and Improves Liver Mass Restoration after Hepatectomy in Mice via Stress-Sensitive Genes

**DOI:** 10.1155/2016/9191407

**Published:** 2016-01-26

**Authors:** Jelena Marinić, Dalibor Broznić, Čedomila Milin

**Affiliations:** Department of Chemistry and Biochemistry, Faculty of Medicine, University of Rijeka, Braće Branchetta 20, 51 000 Rijeka, Croatia

## Abstract

Polyphenols can act as oxidants in some conditions, inducing redox-sensitive genes. We investigated the effect of preexposure to the olive oil polyphenols extract (PFE) on time-dependent changes in the hepatic oxidative state in a model of liver regeneration—a process in which oxidative stress associated with the metabolic overload accounts for the early events that contribute to the onset of liver self-repair. Liver regeneration was induced by one-third hepatectomy in mice. Prior to hepatectomy, mice were intraperitoneally given either PFE (50 mg/kg body weight) or saline for seven consecutive days, while respective controls received vehicle alone. Redox state-regulating enzymes and thiol proteins along with the mRNA levels of Nrf2 gene and its targets *γ*-glutamylcysteine synthetase and heme oxygenase-1 were determined at different time intervals after hepatectomy. The liver mass restoration was calculated to assess hepatic regeneration. The resulting data demonstrate the effectiveness of preexposure to PFE in stimulating liver regeneration in a model of a small tissue loss which may be ascribed to the transient increase in oxidant load during the first hours after hepatectomy and associated induction of stress response gene-profiles under the control of Nrf2.

## 1. Introduction

The liver is endowed with the endogenous mechanism of self-repair which restores the functionality of the organ after injury or surgical resection. This repair process, known as liver regeneration, is mainly divided into two distinctive stages. The first stage is the priming phase, during which quiescent hepatocytes sensitized by cytokines and hormonal and nutritional signals acquire the competence to the cell cycle. The second stage is the proliferating phase, during which cells sequentially transit the G1, S, G2, and M phases, dividing until the original liver mass is restored [1, 2].

Liver resection still remains a common practice in the management of patients with hepatocellular carcinoma [3]. Nevertheless, inadequate hepatic regeneration is still an important cause of morbidity and mortality [4]. Consequently, there is a high interest in the possibility of stimulating this hepatic endogenous mechanism of self-repair to increase survival rates and recovery of patients suffering from hepatic injury of various etiologies. Partial hepatectomy in rodents is commonly used model of liver regeneration which enables quantitative study of the influence of various substances on the liver repair [5]. Although dietary vitamins and plant compounds affect the redox state-regulating enzymes and thiol proteins which are necessary for a successful cell cycle progression and normal growth control [6–8], conflicting impact on liver regeneration was established, involving both impaired [9, 10] and reinforced regenerative response [11]. More recently, diverse classes of plant polyphenols, including those inherent to the olive oil, have been shown to regulate repair-related processes in several wound healing models [12–15]. Moreover, recent developments in oncologic surgery suggest the use of intraperitoneal chemotherapy for the treatment of some human cancers [16, 17]. Besides, intraperitoneal administration has been previously used in animal models [18, 19] and quercetin administered intraperitoneally increased the total quercetin level in tumor tissues more than oral quercetin [20]. Taking into account that the reported effects of olive oil feeding and administration by gavage on antioxidant defenses and liver regeneration have been previously described [21, 22], we aimed to investigate the effect of intraperitoneal administration of polyphenols extract from the olive oil before hepatectomy. Olive oil based emulsions significantly improved the hepatic regeneration and decreased oxidative damage following hepatectomy. Although it is emphasized that this effect is due to the influence of antioxidants present in olive oil, the contribution of polyphenols to the endogenous antioxidant protection system and the healing process has yet to be determined.

The efficacy of polyphenols in wound healing may be ascribed to their purported antioxidant properties, which are assumed to reside in their ability to scavenge different physiologically relevant radical species and to chelate transition metal ions involved in the generation of reactive oxygen species (ROS) [23–25]. However, compiling body of evidence suggests that protective effects of dietary antioxidants from ROS induced injury are not related to their direct antioxidant activity but rather are due to their interaction with specific aspects of the signal transduction network with the ultimate outcome in the modulation of endogenous antioxidant defenses [26–29].

Redox-sensitive transcription factor nuclear factor-erythroid 2-related factor (Nrf2) is central to coordinating expression of genes encoding antioxidant and detoxification enzymes, xenobiotic transporters, and many other proteins involved in the regulation of cell cycle and cell death [30–32]. Studies in Nrf2-deficient mice demonstrated reduced expression of ROS-detoxifying enzymes after hepatectomy, resulting in oxidative stress in hepatocytes, enhanced hepatocyte apoptosis, and a delay in liver regeneration following hepatectomy [33]. In human lung cancer cell lines, growth inhibitory properties of Nrf2 deficiency have been attributed to the induction of cell cycle arrest at G1 phase [34] while, in primary epithelial culture cells, genetic disruption of Nrf2 impaired GSH-induced redox signaling and prevented the progression along the cell cycle, through G2 into M phase [35]. Since cell cycle regulatory machinery induced by hepatectomy is linked to the Nrf2 signaling machinery, there is a possibility that redox-active substances—such as polyphenols—may affect hepatic oxidative state leading to a different course of liver regeneration.

In view of this potential of phenolics, we obtained the polyphenols extract (PFE) from the olive oil and examined its effect on the antioxidant status during the course of liver regeneration following one-third hepatectomy in mice. Although it is well known that antioxidant enzymes are induced by nuclear translocation of Nrf2, [36–38] we evaluated also a possible modulation of its mRNA expression. For that purpose, the changes in the redox state-regulating enzymes, thiol proteins, and Nrf2 gene signatures that coordinate adaptive stress response were determined at 0 (controls), 1, 3, 6, 12, 24, and 48 hours after hepatectomy.

## 2. Materials and Methods

### 2.1. Preparations of Olive Oil Polyphenols Extract (PFE)

Olive oil polyphenols extract was prepared from the olive oil obtained from a mixture of olive cultivars grown in the Island of Krk and the oil was purchased from an individual producer. The oil was produced by a process of continuous centrifugal extraction in three phases and was kept in dark glass bottles in a cool place until analysis. Polyphenols were isolated from the olive oil by liquid-liquid extraction using a methanol-water mixture (60 : 40; v/v) according to Swain and Hillis [39]. The total polyphenol content was determined with Folin-Ciocalteu's reagent (Fluka, Buchs, Switzerland) according to Gutfinger [40], and sodium molybdate solution (Fluka, Buchs, Switzerland) was used to establish the content of* ortho*-diphenols as described by Mateos et al. [41].

For the animal experiments, the methanol-water phase was evaporated to dryness under reduced pressure in a rotary vacuum evaporator (Büchie R-3000 with Büchie Vacuum Controller B-720, Switzerland) at 40°C, and under a nitrogen stream. The dry residue was then dissolved in a physiological saline solution (NaCl 0.9%). Resulting PFE was stored at −20°C in aliquots and used as per requirement.

### 2.2. Animal Experiments

Male C57BL/6 mice, 2-3 months old, with weight 24–30 g, were obtained from the breeding colony of the School of Medicine, University of Rijeka. The animals were housed in propylene cages and maintained in a germ-free environment, at 12-hour light/dark cycle, and constant temperature (20 ± 1°C) and humidity (50 ± 5%), with free access to tap water and a standard rodent diet (pellet, type 4RF21 GLP, Mucedola, Italy). All experimental procedures were conducted in compliance with the Declaration of Helsinki principles and approved by the Ethical Committee of the School of Medicine, University of Rijeka.

Experimental animals were randomly assigned into four groups, with six animals per group as follows: PSS + pHx-mice pretreated with physiological saline solution prior to partial hepatectomy, PFE + pHx-mice treated with olive oil polyphenols extract followed by partial hepatectomy, PSS, and PFE-mice receiving vehicle alone (controls), that is, physiological saline solution or olive oil polyphenols extract.

Physiological solution (0.1 mL per 10 grams of body weight) and a dose of 50 milligrams of the PFE per kilogram body weight (50 mg/kg btw) were intraperitoneally (i.p.) injected daily for seven successive days. The dose of olive oil polyphenols extract used in animal experiments was selected on the basis of our previous study [42] and according to the dose levels reported for oleuropein [43] and its derivative hydroxytyrosol [44, 45], which are the most prevalent phenolic compound in olive oil and the most representative olive oil* ortho*-diphenols [46].

On the eighth day animals receiving vehicle alone underwent sacrifice while animals of the first and the second group were subjected to the 1/3 hepatectomy under appropriate ether anesthesia and in compliance with the rules of asepsis. 1/3 hepatectomy was accomplished by removing the median liver lobe through a midabdominal incision, according to the method described by Beer et al. [47]. In order to avoid the potential impact of circadian rhythm, all operations were performed at the same time of the day, between 8.00 a.m. and 9.00 a.m. Experimental animals were allowed to recover on a 37°C warm plate and then underwent sacrifice at 1, 3, 6, 12, 24, and 48 hours after the surgery. Animals in all experimental groups were sacrificed by cervical dislocation in ether anesthesia.

Before sacrificing, blood was sampled under ether anesthesia by orbital sinus puncture. Whole blood samples were left to stand for two hours at 25°C and then centrifuged at 2.000 ×g over 20 minutes. Obtained serum was stored at −80°C for subsequent analysis of glutathione* S*-transferase alpha.

Upon sacrifice at indicated times, the liver was removed and repeatedly washed in phosphate buffer saline (PBS, Sigma, St. Louis, MQ, USA) to remove blood clots, and total liver weight (controls) and weight of the liver remnant were obtained. Portions of liver tissue masses of up to 100 mg were exempted for RNA extraction while the remaining tissue was used for the analysis of antioxidant enzymes activity, TBARS levels, and the concentration of total glutathione. Tissue samples were immediately weighed, quick-frozen in liquid nitrogen, and stored at −80°C until further processing.

### 2.3. Serum Glutathione* S*-Transferase Alpha (*α*-GST) Level

Serum level of *α*-GST was measured spectrophotometrically using standard sandwich enzyme-linked immunosorbent assay (ELISA) (Oxford Biomedical Research, Inc., Oxford, MI, USA) on a Bio-Tek ELx808 Ultra Microplate Reader (BioTek Instruments, Winooski, VT, USA) following the manufacturer's instructions. The quantity of an enzyme bound to the immunosorbent was measured at 450 nm, and the results are expressed as nanograms per milliliter of the serum (ng/mL).

### 2.4. Hepatic Antioxidant Status

Lipid peroxidation was detected in a 25% liver tissue homogenate prepared in PBS by monitoring pink-colored adduct at 534 nm, which is formed in the reaction with thiobarbituric acid (TBA, Sigma, St. Louis, MQ, USA) under high temperature (95°C) and acidic conditions (pH = 3.5) [48]. The hepatic lipid peroxidation was expressed as TBARS content and was calculated as nanomoles per milligram protein (nmol/mg) according to the standard curve prepared from 1,1,3,3-tetraethoxypropane (Sigma, St. Louis, MQ, USA).

Total glutathione concentration was determined in liver tissue lysates, prepared by tissue homogenization in 5%* meta*-phosphoric acid (w/v) (ACS, Sigma-Aldrich GmbH, Taufkirchen, Germany) using a commercially available HT Glutathione Assay Kit (Trevigen, Helgerman Ct, Gaithersburg, USA), according to the manufacturer's instructions. The quantity of glutathione was established by the optimized enzymatic recycling method with glutathione reductase at 405 nm on a Bio-Tek ELx808 Ultra Microplate Reader (BioTek Instruments, Winooski, VT, USA). The results are expressed as nanomoles of GSSG (equivalent to total glutathione) per gram of liver tissue (nmol/g).

Superoxide dismutase (SOD), glutathione peroxidase (GPx), and glutathione reductase (GR) activities were measured by commercially available kits from Trevigen (Helgerman Ct, Gaithersburg, USA). Cayman Chemical Company kit (Ann Arbor, MI, USA) was used for catalase (CAT) activity determination. Enzyme activities were measured on a Bio-Tek ELx808 Ultra Microplate Reader (BioTek Instruments, Winooski, VT, USA) according to the manufacturer's protocols.

SOD activity (representing the activity of SOD1, SOD2, and SOD3 isoenzymes) was determined in the cytosolic tissue extract from percent inhibition of the rate of formation of tetrazolium salt upon superoxide anion radicals generation in the xanthine/xanthine oxidase system, and it was monitored by measuring the decrease in absorbance values at 450 nm. The results are given in units of SOD per microgram of protein (U/*μ*g), where one unit is defined as the amount of sample that inhibits the rate of increase in absorbance due to the tetrazolium salt formation by 50%.

CAT activity was measured in tissue homogenate by monitoring the decrease in absorbance at 540 nm due to the hydrogen peroxide consumption. The results are given in units of CAT per microgram of protein (U/*μ*g). One unit is defined as the amount of enzyme that will cause the formation of 1.0 nmol of formaldehyde per minute at 25°C.

GPx activity is measured in a tissue homogenate, in a reaction coupled with glutathione reductase by monitoring a decrease in absorbance at 340 nm. The results are expressed in units of GPx per milligram of protein (U/mg). One unit of glutathione peroxidase is defined as the amount of enzyme that will cause the oxidation of 1 nmole of NADPH to NADP^+^ per minute at 25°C.

GR activity is determined in cytosolic tissue extract by measuring the rate of NADPH oxidation. The accompanying decrease in absorbance was monitored at 340 nm. The results are expressed as milliunits of GPx per milligram of protein (mU/mg). One unit of glutathione reductase oxidizes 1 *μ*mol of NADPH per minute at 25°C, pH 7.5.

Protein content was estimated by Bradford's method, with bovine serum albumin (Sigma, St. Louis, MQ, USA) used as a standard [49].

### 2.5. RNA Extraction

Total intact hepatic RNA was extracted using TRI Reagent Solution (Applied Biosystems/Ambion, Foster City, CA, USA) and isolated RNA was purified with RNeasy Mini Kit (Qiagen, Germany) as it is described by the manufacturer. The total RNA concentration was measured based on *A*_260_ value. The purity of the RNA in each sample was verified by determining *A*_260_/*A*_280_ ratio. The integrity of the RNA molecule as well as the separation efficiency was confirmed by 1% agarose-formaldehyde gel electrophoresis with ethidium bromide staining. The samples in which there were clearly visible ribosomal bands, 28S and 18S, with the ratio of upper (28S) and lower (18S) band of about 2 : 1 and in which there was no apparent contamination of genomic DNA were used for further analysis.

### 2.6. Quantitative Real-Time PCR Assays

Single-strand cDNA was synthesized from 5 *μ*g of total RNA in a 50 *μ*L reaction volume following the protocol of High Capacity cDNA Reverse Transcription Kit with RNAse inhibitor (Applied Biosystems, USA). To preclude contamination with genomic DNA, negative controls in which the reverse transcriptase was replaced with water were used and subjected to analogous procedures and conditions. Quantitative real-time polymerase chain reaction (qPCR) was performed using the Power SYBR Green PCR Master Mix (Applied Biosystems, Foster City, CA, USA) and optimized oligonucleotide primers. Primers specific for the mouse genes Nrf2, HO-1, GCSc, and 18S RNA were used. Primers are commercially available as QuantiTect Primer Assay (Mm_Nfe2l2_1_SG for Nrf2, Mm_Hmox1_1-SG for HO-1, Mm_Gclc_1_SG for GCSc, and Mm_Rn18s_2_SG for 18S RNA, Qiagen, Germany). Each reaction was carried out using twenty-five times diluted cDNA product in a 25 *μ*L reaction volume. Two replicates of each reaction were performed. PCR amplification was conducted on Real-Time PCR 7300 (Applied Biosystems, Foster City, CA, USA) and the amplification conditions were 50°C for 2 min, 1 cycle; 95°C for 10 min, 1 cycle; 95°C for 15 sec, 40 cycles; and 60°C for 1 min. Amplicon specificity was verified by first-derivative melting curve analysis for each pair of primers. The expression levels of the genes were normalized to that of the housekeeping gene, 18S RNA, and were calculated using the standard 2^−ΔΔCT^ method. The amount of transcript expressed at time zero (vehicle controls) was used as calibrator sample.

### 2.7. Calculation of Liver Mass Restoration

Surgical procedure resulted in removal of approximately 1/3 of the total liver mass while 2/3 of the remaining liver exerted the compensatory growth. The intensity of that regeneration in the group of mice that received saline or polyphenols extract before surgery (experimental groups) was expressed as a percentage of recovery of excised liver weight in relation to the estimated liver weight at the operation and as a function of time after hepatectomy. The calculations of liver mass restoration were made according to following formula: (actual mass of the liver remnant at a given time − expected mass of the liver remnant immediately after the liver resection)/(prehepatectomy liver mass) × 100. Expected mass of the liver remnant immediately after the resection was calculated as [(actual mass of the liver remnant in experimental group)/(body mass in experimental group)]/[(liver mass in control group)/(body mass in control group)]. Prehepatectomy liver mass was quantified as follows: [(liver mass in control group)/(body mass in control group)] × (body mass in experimental group).

### 2.8. Statistical Analysis

The data were analyzed using StatSoft STATISTICA version 12 software. Normality of data distribution was assessed by the Kolmogorov-Smirnov normality test. The distribution qualified the normality test, so parametric test was applied. For comparison between groups, the unpaired Student's *t*-test (in case of only two comparisons) or analysis of variance (one-way ANOVA) was used. Fischer post hoc test was applied when variances across the group were equal, and Dunnett's post hoc test was used when variances were not equal. Variance equality was tested by Levene statistical analysis. Results are expressed as mean ± standard deviation (SD). Differences with *p* < 0.05 were considered to be statistically significant.

## 3. Results

### 3.1. Extract Composition

The total polyphenol content was 326.23 ± 4.27 mg/kg expressed as gallic acid equivalents and the* ortho*-diphenols content was 22.46 ± 0.39 mg/kg expressed as caffeic acid equivalents.

### 3.2. Hepatic Lipid Peroxidation

**Figure 1:**
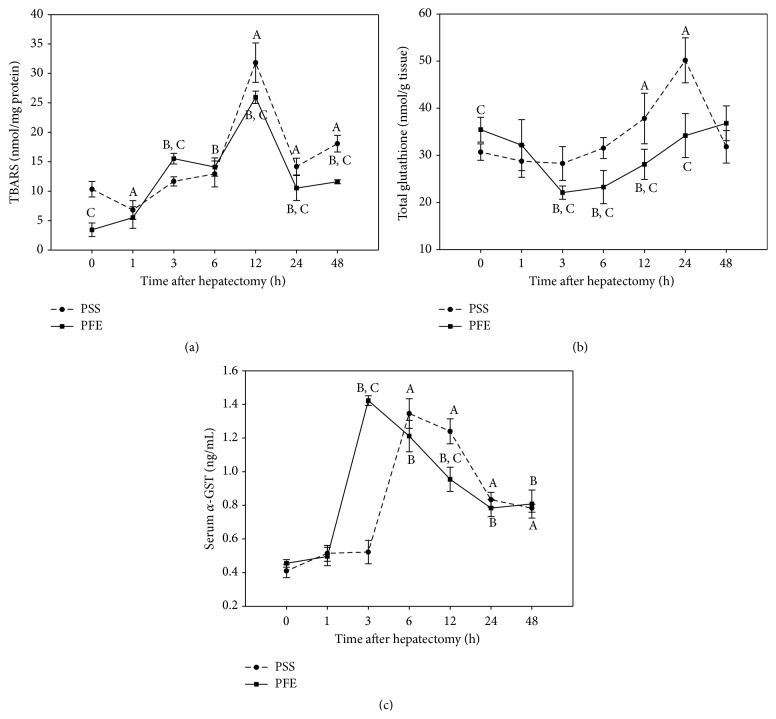
(a) Hepatic TBARS level, (b) total liver glutathione content, and (c) serum glutathione *S*-transferase level in the polyphenols extract treated and untreated hepatectomized mice at different time intervals after the surgery. Time zero correspondents to values from the unhepatectomized, vehicle (PSS or PFE) treated mice. Results are the mean ± SD of six individual determinations for each experimental point, *p* < 0.05; (A) significantly different from the unhepatectomized mice receiving PSS at a given time point; (B) significantly different from the unhepatectomized mice receiving PFE at a given time point; (C) significant difference between PFE treated and untreated hepatectomized mice at a given time point.

The amounts of TBARS were determined to assess lipid peroxidation level. Initially, untreated animals exhibited a significantly higher level of TBARS (Figure 1(a)), but in both experimental groups substantial enhancement of lipid peroxidation was recorded, reaching a peak at 12 hours after the surgery. However, polyphenols induced marked increase in TBARS levels at earlier times (3 hours after hepatectomy). Although the content of TBARS did not regain initial level until the end of the experimental period in treated experimental group, time intervals following 6 hours after hepatectomy were characterized by a small, but significant, attenuation of lipid peroxidation in polyphenols treated and hepatectomized mice versus mice subjected only to hepatic resection.

### 3.3. Hepatic Glutathione Content

Hepatic resistance towards oxidative injury was determined by assessing the total hepatic glutathione content (comprising reduced GSH and oxidized GSSG form). Partial hepatectomy alone did not induce a significant alteration in total hepatic glutathione content (Figure 1(b)) until 12 hours after the surgery when it showed significant increase with respect to the initial values which is retained for up to 24 hours after hepatectomy. In spite of the initially higher values, PFE treatment promoted a sharp drop in total glutathione at 3 hours after hepatectomy compared to the group of untreated hepatectomized mice and in relation to the initial values, reflecting increased oxidant load. Although the concentration of the total glutathione showed increasing course in subsequent time intervals in PFE treated and hepatectomized mice, the level of this nonenzymatic antioxidant remained significantly lower compared to the untreated group up to 24 hours after hepatectomy.

### 3.4. Serum *α*-GST Level

To determine the capacity of detoxification of lipid peroxidation end products and various electrophilic molecules in the regenerating liver, serum *α*-GST level was assessed. The level of *α*-GST (Figure 1(c)) was significantly elevated in both experimental groups, although in PFE treated animals it peaked earlier (3 hours after hepatectomy) and was promptly reduced in magnitude but not significantly changed in relation to the untreated animals, except at 12 hours postoperatively, reflecting lower oxidant/electrophilic load in mice receiving PFE compared to the untreated animals. Although in periods after the maximum *α*-GST level tends to decrease, detoxification demands still remained high in the regenerating liver of both experimental groups, since the level of *α*-GST was not fully recovered until the end of the experimental period compared to the initial values of respective control groups.

### 3.5. Activities of the Thiol Modulating Enzymes (GPx, GR) and Antioxidant Enzymes (SOD, CAT)

In addition to glutathione, we determined the functionality of the glutathione-dependent enzymes and other antioxidant enzymes that make up the first and second line of defense against the cytotoxicity of ROS and electrophiles.

**Figure 2:**
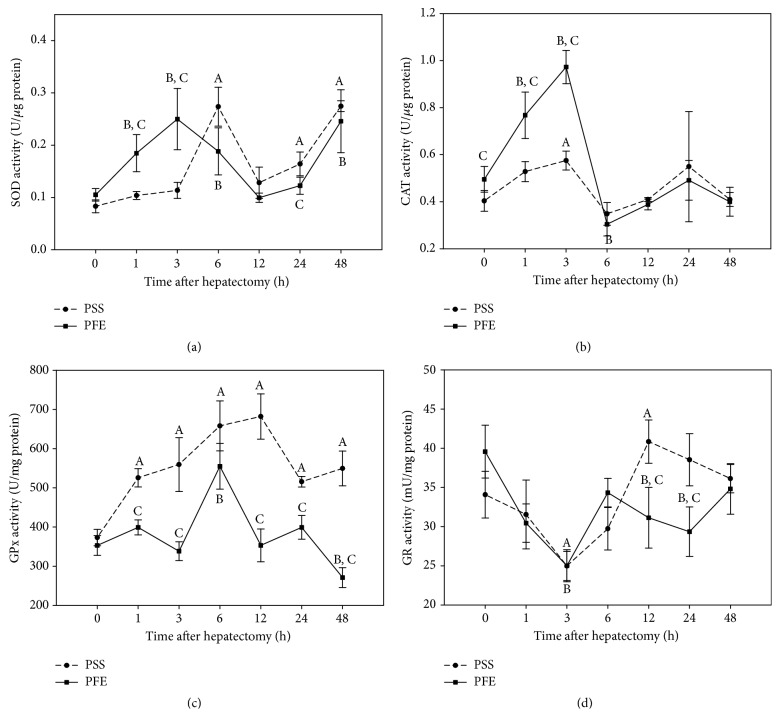
(a) Superoxide dismutase, (b) catalase, (c) glutathione peroxidase, and (d) glutathione reductase activity in the polyphenols extract treated and untreated hepatectomized mice at different time intervals after hepatectomy. Time zero correspondents to values from the unhepatectomized, vehicle (PSS or PFE) treated mice. Results are the mean ± SD of five to six individual determinations for each experimental point, *p* < 0.05; (A) significantly different from the unhepatectomized mice receiving PSS at a given time point; (B) significantly different from the unhepatectomized mice receiving PFE at a given time point; (C) significant difference between PFE treated and untreated hepatectomized mice at a given time point.

Figure 2(a) denotes a gradual increase in SOD activity, in the liver tissue of both experimental groups, reflecting greater demands for the superoxide anions detoxification. However, polyphenols shifted the activity maximum towards the earlier time periods (3 hours after hepatectomy) which was also accompanied by the depletion of cellular glutathione, increased lipid peroxidation products, and electrophiles generation. After a transient decline in SOD activity, which in the treated group starts earlier, the relation of the SOD activities in two experimental groups changes, showing a more pronounced increase in the nontreated hepatectomized group. However, 48 hours after hepatectomy, there was no significant difference in SOD activity in hepatectomized mice receiving PFE and mice receiving vehicle.

Cytotoxicity of hydrogen peroxide, generated during the catalytic cycle of SOD, is counterbalanced by the CAT and GPx activities. These enzymes are characterized by different *K*_*m*_ values for their substrate, and their activity shows an inverse relationship in the treated versus untreated mice. In the group of untreated mice, CAT activity (Figure 2(b)) is close to the initial value until the end of the experimental period. A low CAT activity reflects the lower concentration of hydrogen peroxide. Under these conditions, this nonradical reactive oxygen species is detoxified by the GPx. GPx activity (Figure 2(c)) shows a continuous upward trend after hepatectomy in the untreated group and remains elevated until the end of the monitoring period (48 hours after hepatectomy). On the other hand, the polyphenols treatment is reflected in the increase in CAT activity (Figure 2(b)), which peaked at the third hour after hepatectomy. At this time point, SOD activity displays a maximum and glutathione is markedly depleted, indicating the greater extent of oxidative stress in the regenerated liver of treated mice when compared to the untreated mice subjected to the operative procedure. This marked increase is followed by a temporary decline and activity normalization at 12 postoperative hours. In subsequent time intervals SOD activity increases in PFE pretreated mice and displays significantly lower values at 24 hours after hepatectomy in relation to untreated mice. At the same time, GPx activity (Figure 2(c)) was not significantly changed compared to the initial values, except at the 6 hours after hepatectomy, when it shows an increase, most likely to compensate for reduced CAT activity (Figure 2(b)) to counteract peroxides formation, but at the 48 hours GPx activity displays marked activity reduction (Figure 2(c)). Moreover, PFE pretreatment markedly decreased GPx activity in comparison to untreated mice in periods from 12 to 48 hours after hepatectomy.

Processes of glutathione utilization result in the formation of its oxidized GSSG form. To determine the capacity of glutathione recycling back to its reduced form, GR was assessed. GR activity (Figure 2(d)) was not significantly changed in the regenerating liver tissue of both experimental groups, except at 3 hours postoperatively in both groups, where GR lower activity denotes impaired glutathione recycling in treated and untreated mice. However the hepatic capacity of glutathione regeneration is restored at 12 hours after hepatectomy, but only in the nontreated group, reflecting the increase in glutathione level. Although GR activity showed increasing trend upon polyphenols treatment following 24 hours after hepatectomy, it remained lower compared to the initial values but not significantly different compared to the untreated group. Furthermore, significantly lower GR activity was observed at 12 and 24 hours in the PFE treated hepatectomized versus untreated hepatectomized mice.

### 3.6. Gene Expression Profile

**Figure 3:**
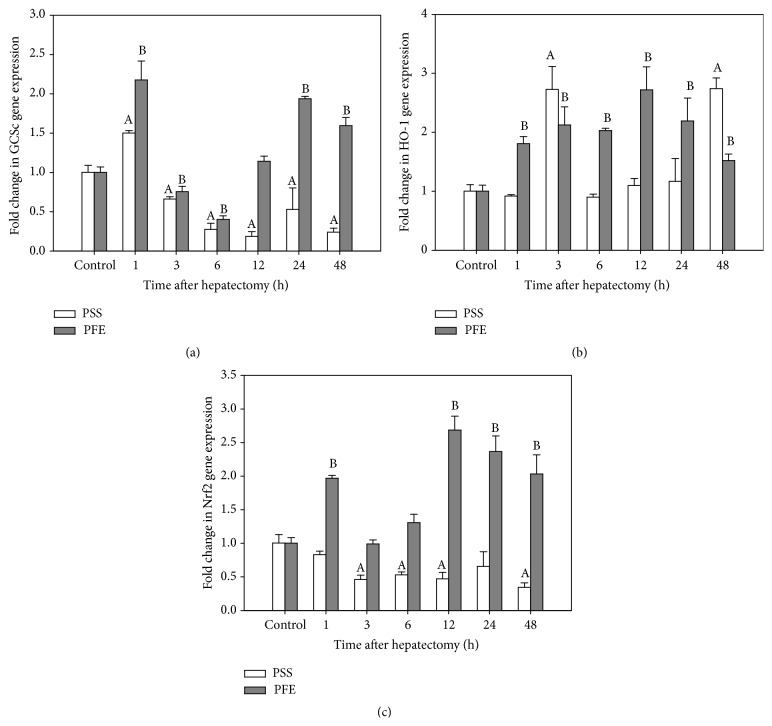
Real-time quantitative PCR analysis of (a) GCSc gene, (b) HO-1 gene, and (c) Nrf2 gene expression levels in a regenerating liver at different time intervals after hepatectomy. Total RNA, isolated from the liver of three animals, was pooled, subjected to a reverse transcription, and the resulting first-strand cDNA was amplified using primers specific for the mouse GCSc, HO-1, and Nrf2 gene. The results are expressed as the relative ratio of each cDNA level over vehicle control (PSS or PFE) after normalization to corresponding 18S RNA levels. Data represent mean ± SD of five to six animals per group (*p* < 0.05), and each determination was conducted in duplicate; (A) significantly different from the PSS vehicle control mice at a given time point; (B) significantly different from the PFE vehicle control mice at a given time point.

In order to establish at what stage PFE exert their glutathione increasing activity, mRNA level of the *γ*-glutamylcysteine synthetase catalytic subunit (GCSc) was analyzed by quantitative PCR. *γ*-Glutamylcysteine synthetase is the key determinant of the overall glutathione biosynthetic capacity, catalyzing the rate-limiting step of this metabolic reaction. The enzyme is composed of heavy (catalytic) and light (regulatory) subunit, with the former being responsible for all of the catalytic activity, including a feedback inhibition by glutathione [50]. The immediate early period (1 hour) after hepatectomy is marked by the initial rise in GCSc gene transcriptional activity (Figure 3(a)) in both experimental groups. In this period, the level of mRNA GCSc genes in the liver of treated group was approximately two times higher and one and a half times higher in the untreated group when compared with respective controls. After that a gradual decline followed in the level of GCSc transcript, which is detained in the untreated group by the end of the experimental period and, therefore, the established increase in the concentration of glutathione is not a reflection of the increased capacity of its biosynthesis. By contrast, in the treated group at 12 hours postoperatively reinduction of GCSc gene expression was observed that lingers until the end of the experimental period, coinciding with periods in which increase in the concentration of glutathione in this group was found.

Another important cytoprotective gene with the role in cell proliferation is HO-1 [51]. Thus, RT-PCR was employed to establish whether the PFE pretreatment exerts the induction of HO-1 at the transcriptional level. In regenerating liver tissue of PFE treated group, a significant and relatively stable increase in expression of HO-1 gene (Figure 3(b)) in all time intervals after hepatectomy was accomplished, except at 48 hours after the surgery. Transcriptional activity of HO-1 gene in the untreated group was suppressed in all periods, except at 3 and 48 hours after the operation, when approximately two and half times higher level of HO-1 mRNA transcript was accomplished in comparison to the controls.

Promoter regions of the GCSc and HO-1 gene contain common consensus elements which bind Nrf2 transcriptional factor [52, 53]. Therefore, we next seek to examine the effects of PFE pretreatment on the Nrf2 induction at the transcriptional level. RT-PCR, using Nrf2-specific primers, revealed suppression of the Nrf2 gene transcriptional activity (Figure 3(c)) in all periods after hepatectomy. In contrast, polyphenols treatment induced Nrf2 gene expression at 1 hour after hepatectomy, when approximately two times higher level of Nrf2 gene transcript was accomplished in comparison to the control group. Three hours after the surgery transcriptional activity was slightly suppressed but at 6 hours started to increase and at 12, 24, and 48 hours reached the value of about 2–2.5 times higher than the control.

### 3.7. Liver Mass Restoration

**Figure 4:**
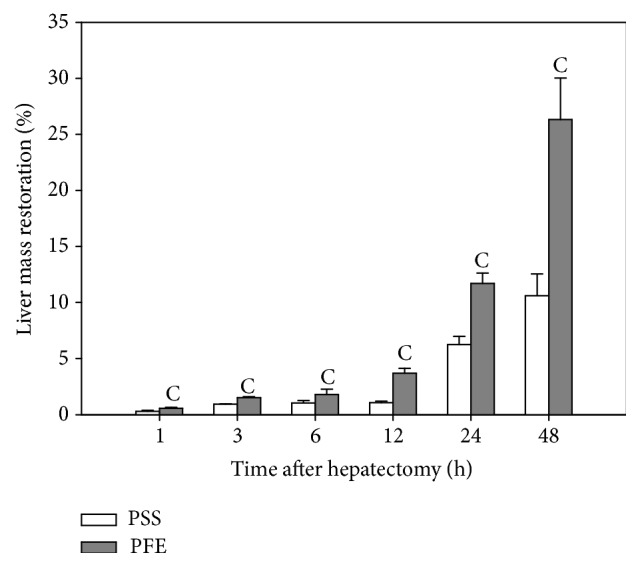
The restoration of the initial liver mass in a function of time after hepatectomy. Regeneration rate of the remnant liver tissue was expressed as the percentage of the restoration of the excised liver mass in relation to the estimated prehepatectomy liver mass at a given time; (C) significantly different from the vehicle control group (*p* < 0.05).

In both experimental groups, the gradual increase of recovered liver mass was observed throughout the entire experimental period (Figure 4). However, the polyphenols treatment manifested itself in the significantly higher intensity of that regeneration in all time intervals after hepatectomy. At the end of the experimental period, approximately 15% higher regeneration ratio of the remnant liver tissue was recorded in a group of mice receiving PFE compared to those that underwent only hepatectomy.

## 4. Discussion

Several studies addressing the role of polyphenols in tissue repair attributed wound healing potential of these plants derived compounds to their antioxidant capacity based on the observation that ROS, produced in response to tissue injury, impede or exacerbate the healing process. We investigated the effect of preconditioning with PFE on the course of liver regeneration induced by hepatectomy—a process during which ROS account for the early signals involved in the initiation of tissue mass restoration.

The results of the present study indicate that regenerating liver itself exerts intrinsic oxidative stress. Pretreatment with PFE additionally elevated the overall oxidant load within the first three hours after hepatectomy. This stage corresponds to the priming phase of liver regeneration, which increases the sensitivity of hepatocytes to growth factors leading up to DNA replication and mitosis [1]. Initial oxidative stimuli provoked by polyphenols promoted depletion of total glutathione, one of the major determinants of the hepatic resistance towards oxidative injury, and alterations in processes linked to the glutathione pathway. These processes entailed the impaired glutathione redox cycling due to the decreased GR activity, impaired glutathione biosynthesis as indicated by suppressed GSCc transcriptional activity, and increased glutathione utilization via the GST-mediated reactions. Glutathione depletion coincides with the period in which activity of enzymes that first confront the cytotoxic effects of ROS, that is, SOD and CAT, reached peak values. These antioxidant enzymes afforded only partial protection against hydrogen peroxide, superoxide anion radical, and hydroxyl radical as noted by the simultaneous increase in lipid peroxidation level and a peak in serum *α*-GST enzyme. It has been demonstrated that *α*-GST serum levels reflect induction of hepatic *α*-GST in mice when fed with phase II enzyme inducers [54]. Induction of GSTs has been described for phenols sharing 1,2-diphenol structure [55] and plays important roles in the detoxication of electrophiles generated by phase I enzymes and lipid peroxidation end products. Inducers of GSTs are also substrates for GSTs and, in addition, have the ability to elevate tissue glutathione levels [24]. This standpoint is substantiated in our model where a general trend upward was noted in glutathione levels upon PFE pretreatment in periods prior to the onset of DNA synthesis (between 6 and 24 hours after hepatectomy). Concurrently with the increase in glutathione content, *α*-GST and lipid peroxidation level gradually decline, while the activities of redox state and thiol regulating enzymes return to the basal level which is in accordance with the hypothesis that oxidative stress is reduced before cell division [56]. However, periods coinciding with replicative phase (48 hours after hepatectomy) are characterized by an increase in SOD activity in both experimental groups, which is considered to be a measure of protection against ROS generated due to increased oxidative metabolism in the S phase of the cell cycle [57]. This time point is also characterized by decreased GPx activity in mice receiving PFE, probably due to the inhibition of GPx by nitrogen (II) oxide which rises immediately after hepatectomy and exerts stimulating effects on liver regeneration [58].

Although lipid peroxidation coupled with glutathione depletion can represent one of the mechanisms of cell toxicity, selective enhancement of lipid peroxidation can act as potential mediator of early regenerative capacity by evoking a general cell response involving activation of transcriptional factors [10].

In mammalian cells, low levels of oxidative stress initiate activation of prosurvival transcription factor Nrf2 which is indispensable for a successful cell cycle progression and coordinated induction of battery cytoprotective genes [30, 34, 35], including GCSc and HO-1 [52, 53] which are known to be associated with the control of liver regeneration.

Our results suggest that oxidative stress induced by polyphenols pretreatment elevated mRNA of GCSc and HO-1 genes and increased Nrf2 gene expression.

Based on the observation that decreased expression of Nrf2 target genes results in extensive oxidative stress and in a delay in liver regeneration in Nrf2^−/−^ deficient mice [33, 59], it seems reasonable to conclude that induction of GCSc and HO-1 genes upon polyphenols pretreatment is related to the cellular adaptation to oxidative stress and exerts stimulatory role in liver regeneration.

The ability of polyphenols to induce GCSc and HO-1 mRNA expression has been confirmed in both* in vivo* and* in vitro* models [8, 26, 60], and both of the genes were upregulated in response to PFE pretreatment.

The induction of GCSc gene by PFE is accompanied by the continuous increase in total glutathione concentration, necessary for stimulation of the hepatocytes proliferative response and entry into the S phase of the cell cycle [50]. However, in spite of these changes the concentration of total glutathione remained lower compared to the untreated animals, possibly due to the enhanced biliary glutathione excretion which has been observed to occur as a result of Nrf2-induced GST mRNA expression and enzyme activity [61], as well as the HO-1 induced CO formation [62]. Both HO-1 and its metabolic product CO have a pivotal role in the liver regeneration after resection. Activation of HO-1 in hepatocytes is directly associated with the release of NO, small gas signal transduction molecule which provides protection against apoptosis and favors progression in the cell cycle until the organ size and function are restored after partial hepatectomy [58]. Elevated expression of HO-1 is typically associated with tissue adaptation and activation of survival pathways and improves hepatic graft function and posttransplantation survival, mostly as a consequence of the antiapoptotic and anti-inflammatory effects of CO arising from heme degradation in hepatocytes [63]. Recently, it has been demonstrated that CO increases HGF growth factor secretion from hepatic stellate cells which accelerates early hepatocyte proliferation, possibly through Akt-cyclin-dependent pathway [51].

Although olive oil phenolics are commonly perceived as substances endowed with antioxidant activity, emerging evidences propose that most dietary antioxidants, with the exception of vitamin E, do not have a direct role in reducing intracellular oxidants [64]. In fact, cell culture studies have highlighted that the most representative olive oil phenols, hydroxytyrosol, as well as oleuropein, at high concentration (100 *μ*M) have the ability to produce hydrogen peroxide [65], while tyrosol potentiates hydroperoxides increase irrespective of the concentration used (10–250 *μ*M) [25].

Extensive TNF*α* signaling and the increased metabolic activity of hepatocytes to maintain homeostasis increase the levels of ROS and oxidative stress [66], shifting intracellular redox status toward a more oxidizing state in the early stages of regeneration [2]. In this context, phenolics, and especially 1,2- and 1,4-diphenols, which are ubiquitously present in the olive oil, can easily undergo (auto)oxidation producing semiquinone or quinone radicals, or they can become radicals themselves during the reaction with free radicals [8, 23], thereby contributing to the overall oxidant/electrophilic load. In most cases, the prooxidant effects of polyphenols are stimulated by the interaction with transition metal ions which catalyze the oxidation of* ortho*- and* para*-hydroquinones to their corresponding quinones and increase superoxide anion radical and hydrogen peroxide formation. The latter can accelerate the generation of highly reactive hydroxyl radicals via Fenton chemistry [67–69]. Certain forms of quinone radicals can act as electrophiles and form conjugates with glutathione thus lowering the cellular glutathione supplies [68]. Recently it has been demonstrated that both hydroxytyrosol and oleuropein at low concentration participate in the initiation process of LDL oxidation induced by Cu^2+^ ions [70]. It may be assumed that interactions with transition metal ions could be important in the regenerating liver since hepatectomy induces early redistribution of iron from the spleen and thymus into the tissue of regenerating liver [71] but also enhances oxygen delivery to the cells, necessary for the oxidative activation of phenolics via the redox cycling mechanism.

Emerging evidence suggests that prooxidant properties and direct interactions with redox-sensing proteins induce antioxidant enzymes and increase their substrates, leading to the maintenance of nucleophilic tone and protection against ROS induced injury under physiological conditions [64]. Central to the regulation of nucleophilic tone is Nrf2 which is under physiological conditions sequestered in the cytoplasm as an inactive complex with its repressor protein Keap1. It has been proposed that quinone derivatives formed by oxidation of diphenols act as activated Michael acceptors, capable of covalently modifying or oxidizing redox-sensitive cysteine thiols in the sensor protein Keap1. This, in turn, results in the disruption of Nrf2/Keap1 complex and increased stability of Nrf2 and its accumulation in the nucleus where it can transactivate ARE/EpRE-driven target genes [27–29].

Alternatively, by lowering the cellular level of GSH, reactive polyphenol derivatives may temporarily disrupt the redox state of the cell and trigger upstream kinases that phosphorylate Nrf2 aiding in its release from Keap1 [32]. Hydroxytyrosol, principal olive oil diphenol, has been shown to activate Nrf2 through the PI3K/Akt and ERK1/ERK2 pathways in vascular endothelial cells [72]. Similarly, Patel and Maru [27] established that pretreatment of mice with polymeric black tea polyphenols extract increased the level of Nrf2 by posttranslational modifications involving upstream kinases in liver and lung cells, but no significant alterations were observed at the transcriptional level. In this study olive oil extract administration was associated with an increase of Nrf2 mRNA expression level. Those differences may indicate differences in the mode of action of different plant extracts as well as differences in the mechanism of Nrf2 induction under different cellular conditions. One limitation of the present study is that we did not measure Nrf2 activation upon olive oil polyphenols before treatment. The relevance of transcriptional modulation as a regulatory mechanism in the action of Nrf2 will be considered in our future work.

In view of these facts, we propose that increase in oxidant/electrophilic load during the early phase of liver regeneration, mediated via polyphenols* per se* or through glutathione depletion, might represent the signaling mechanism through which the induction of Nrf2 dependent gene signatures could be achieved, resulting in timely and adequate appearance of events associated with liver regeneration. Detailed mechanistic studies are needed to improve the understanding of the antioxidant or prooxidant effects of olive oil phenolics in liver regeneration.

## 5. Conclusion

To our knowledge, this study is the first to demonstrate that PFE preexposure affects endogenous cellular defense mechanisms via the stress response gene-profiles associated with hepatoprotection in the model of liver regeneration induced by one-third hepatectomy. Our results suggest that treatment with PFE, prior to hepatectomy, evoked transient and early increase in electrophilic and oxidative load, which was followed by the increase in Nrf2 and Nrf2-dependent gene expression, leading to certain regenerative signals that resulted in the efficient liver mass restoration. Since Nrf2 participates in the maintenance of redox-homeostasis and regulation of cell cycle, it is plausible to believe that a possibility of inducing Nrf2 could represent a foundation for molecular events leading to cytoprotection and more rapid recovery. These findings support the hypothesis that hepatectomies less than 40%, which are generally characterized by a slower regenerative response, can be efficiently modulated by increasing metabolic load before the resection [73]. Furthermore, we demonstrate that olive oil polyphenols can act as oxidants in some conditions and activate protective mechanisms under the control of redox-sensitive genes which could have stimulating beneficial health response during stressful conditions. Specifically, the possibility of enhancing liver mass restoration by polyphenols in cases of small tissue deficit holds a promise of affecting the development of liver regeneration in situations of overly small liver and displays a potential of application in the field of regenerative medicine.
